# Less basal thyrotropin levels predict antidepressant response in patients with major depression

**DOI:** 10.1192/j.eurpsy.2022.662

**Published:** 2022-09-01

**Authors:** R. Navinés, G. Oriolo, M. Mora, M. Cavero, E. Gómez-Gil, R. Martin-Santos

**Affiliations:** 1 Hospital Clínic, 1psychiatry And Psychology, Barcelona, Spain; 2 Hospital Clinic, Endocrinology And Nutrition, Barcelona, Spain; 3 Hospital Clínic, 1department Of Psychiatry And Psychology, Barcelona, Spain

**Keywords:** tirotrophin, major depression, antidepressant response, MADRS

## Abstract

**Introduction:**

The close association among thyroid metabolism, mood disorders and behavior has long been known. The role of basal thyroid axis in antidepressant treatment response is less known.

**Objectives:**

The aim of the present study was to study the association of basal serum thyrotropin (TSH) levels, with antidepressant treatment response in major depressive disorder.

**Methods:**

Thirty-one depressed adult outpatients were included. Major depressive episode was diagnosed through the MINI (DSM-IV-TR) interview. Clinical symptomatology and blood samples were assessed at baseline, and at 4- and 8-weeks of either escitalopram or sertraline. Treatment response was defined by an improvement ≥50% in MADRS scores at 4-, and 8-weeks. Basal TSH levels were included in a linear regression model as predictor of treatment response.

**Results:**

Twenty-seven patients finished 8-weeks of treatment. Response to treatment was of 74% at 4-weeks, and 63% at 8-weeks of antidepressant treatment. Basal median TSH levels were between normal ranges (M+SD=1.85+1,02 mlU/L). Basal TSH levels not correlated with basal MADRS scores, but with higher MADRS scores at week-4 (r=0,415, p=0,031) and at week-8 (r=0,392, p=0,043). Moreover, less baseline TSH levels trend to be a significant good predictor for treatment response at 4-weeks (R^2^=.116, p=.083); and a good predictor at 8-weeks treatment (R^2^=.147, p=.049).

**Conclusions:**

Baseline TSH levels even within the normal range may play a role in predicting antidepressant response.

**Disclosure:**

No significant relationships.

